# All-*trans* Retinoic Acid as a Versatile Cytosolic Signal Modulator Mediated by CRABP1

**DOI:** 10.3390/ijms20153610

**Published:** 2019-07-24

**Authors:** Isha Nagpal, Li-Na Wei

**Affiliations:** Department of Pharmacology, University of Minnesota, Minneapolis, MN 55455, USA

**Keywords:** All-*trans* retinoic acid, CRABP1, RAF-MEK signaling, CaMKII signaling, ESC, NSC, Cardiomyocyte

## Abstract

All-*trans* retinoic acid (AtRA), an active metabolite of vitamin A, is recognized for its classical action as an endocrine hormone that triggers genomic effects mediated through nuclear receptors RA receptors (RARs). New evidence shows that atRA-mediated cellular responses are biphasic with rapid and delayed responses. Most of these rapid atRA responses are the outcome of its binding to cellular retinoic acid binding protein 1 (CRABP1) that is predominantly localized in cytoplasm and binds to atRA with a high affinity. This review summarizes the most recent studies of such non-genomic outcomes of atRA and the role of CRABP1 in mediating such rapid effects in different cell types. In embryonic stem cells (ESCs), atRA-CRABP1 dampens growth factor sensitivity and stemness. In a hippocampal neural stem cell (NSC) population, atRA-CRABP1 negatively modulates NSC proliferation and affects learning and memory. In cardiomyocytes, atRA-CRABP1 prevents over-activation of calcium-calmodulin-dependent protein kinase II (CaMKII), protecting heart function. These are supported by the fact that *CRABP1* gene knockout (KO) mice exhibit multiple phenotypes including hippocampal NSC expansion and spontaneous cardiac hypertrophy. This indicates that more potential processes/signaling pathways involving atRA-CRABP1 may exist, which remain to be identified.

## 1. Introduction

Vitamins are essential micronutrients required for a myriad of biological processes. Vitamin A was first known to play an essential role in vision [1,2]. Its chemical structure was first deciphered in 1931 [3] and later crystallized in 1937 [4]. Since then, numerous studies have demonstrated the significance of vitamin A and its metabolites in various physiological processes such as fertility [5], embryonic development [6,7], and many other biological processes for survival and maintaining health [8,9,10,11,12,13,14,15]. Vitamin A must be provided by one’s diet and is metabolized into different metabolites collectively called the retinoids, including retinoic acid (RA), retinol (ROL), retinal, etc., characterized based on the functional modification concatenated to the extreme end of the core scaffold [13,16]. Amongst these retinoids, various isomeric forms of RA: atRA, 9-*cis*-retinoic acid, and 13-*cis*-retinoic acid are best studied for their activities in regulating gene expression via binding to nuclear RA receptors (RARs) or retinoid X receptors (RXRs) [17,18,19,20]. Together, these mediate the well-established, canonical action of vitamin A as an endocrine factor that mainly acts to provide a homeostatic control of gene expression to maintain health [21,22].

Interestingly, recent studies have begun to demonstrate that vitamin A, especially its most active metabolite, atRA, can also elicit specific non-canonical (RAR/RXR-independent) activities beyond regulating gene expression [23,24,25,26,27]. Using genetic tools, it has been established that most of these non-canonical activities are mediated by a specific, highly conserved, cellular binding protein for atRA named CRABP1 that serves as a cytosolic signal modulator/integrator or functions as an RA-regulated signal scaffold [27]. This review focuses specifically on these new findings and the emerging concept that atRA can exert specific, non-canonical (RAR/RXR-independent) physiological activities through CRABP1.

## 2. Generation of RA

Vertebrates, including humans, derive vitamin A from plant sources as α and β carotenoids (commonly called proretenoid carotenoids) [28,29], and metabolites of vitamin A from animal sources as retinyl esters (REs) or retinol (ROL) [30,31]. In humans, vitamin A is metabolized in the small intestines and stored in the liver [32,33,34]. Carotenoids are metabolized by two pathways: central and eccentric pathways. The central pathway engages β-carotene 15, 15′-Dioxygenase 1 (BCO1) enzyme to cleave the core double bond of β-carotene, resulting in two molecules of retinal (retinaldehyde) [35], which are further reduced to form ROL by ROL reductases [36] to be stored or further metabolized. The eccentric pathway can be triggered enzymatically or non-enzymatically [37] to cleave the non-central β-carotene double bond [38], generating retinoids such as RAR antagonists, β-carotenes, and β-apocarotenones [39]. ROL can be directly absorbed by the mucosal cell of the small intestine [40], while REs are further hydrolyzed for intestinal absorption by enzymes such as pancreatic triglyceride lipase and pancreatic lipase-related protein 2 [32,41]. The absorbed ROL is re-esterified by transmembrane lecithin retinol acyltransferase (LRAT) [42,43] and incorporated into chylomicrons that are transferred to hepatic stellate cells [44,45] for storage or taken up by other organs/tissues [33,46,47].

Circulating ROL is carried by ROL-binding proteins (RBPs), which are then taken up by cells via STRA6 (stimulated by retinoic acid gene 6), a cell surface receptor for holo-RBP [31,48,49,50,51]. Inside the cells, ROL is converted to retinaldehyde by ROL dehydrogenases (RDHs) or retinyl esters [31]. Retinaldehyde is then irreversibly oxidized to RA by retinaldehyde dehydrogenases (RALDHs), which are key enzymes controlling the intracellular RA concentrations [52,53]. For cells that do not generate RA, they can obtain RA from RA-producing neighboring cells as a paracrine or from circulation as an endocrine factor [54]. Inside the cells, RA is first received by one of the two cellular RA-binding proteins (CRABPs) in the cytoplasm: CRABP1 or CRABP2 [55,56]. It is believed that RA, received by CRABP1, is targeted for degradation by cytochrome (CYP26) family members [57,58,59]. Whereas, when bound to CRABP2, RA is delivered to the nucleus for RAR/RXR-mediated regulation of the expression of RA-targeted genes [60,61,62,63]. This RAR/RXR-mediated gene-regulatory activity of RA elicits the well-established canonical signaling of RA and is believed to underlie the ubiquitous importance of vitamin A as an essential nutrient and endocrine factor. Studies of canonical actions of RA have been extensively reviewed; readers are referred to several excellent reviews in the literature for more detail [18,21,60,64,65,66,67,68,69].

## 3. Non-Canonical Activities of atRA

Recent studies have begun to show that RA, especially atRA, can also modulate cellular growth and function without involving RAR or RXR-mediated gene regulation. Most of these activities are specific to the cytoplasm, and they are mostly mediated by CRABP1. While the physiological implications of these findings remain debatable, studies using *CRABP1* gene KO mice and primary cells have presented compelling evidence supporting this emerging concept. One unique feature of these non-canonical activities is their context-dependency, as demonstrated in the context of cellular growth (for stem cells) [26,70,71] and specific cellular function (for differentiated cells such as cardiomyocyte) [72]. These non-canonical functions of atRA are reviewed in the following.

### 3.1. atRA-CRABP1 Cross-Talks with RAF-MEK-ERK to Dampen Stem Cell Growth

The first evidence of the non-canonical activity of atRA was presented in studies using ESC, which is used to modulate growth factor sensitivity, as reflected by the activation of extracellular signal-regulated kinases 1/2 (ERK1/2) [73,74]. ERK1/2 is a known atRA-regulated gene in ESC; its gene expression is typically up-regulated by atRA approximately 8–12 h post-RA addition. However, careful kinetic studies revealed a surprising biphasic (15 min and 8 h, respectively) activation of ERK1/2 by atRA in ESC. Intriguingly, while phase 2 activation (8 h post-treatment) is effectively blocked by RAR antagonists (indicated via the canonical activity of RA), phase 1 (15 min post-treatment) activation cannot be blocked by RAR antagonists, suggesting a non-canonical response to RA [23,26].

**Figure 1:**
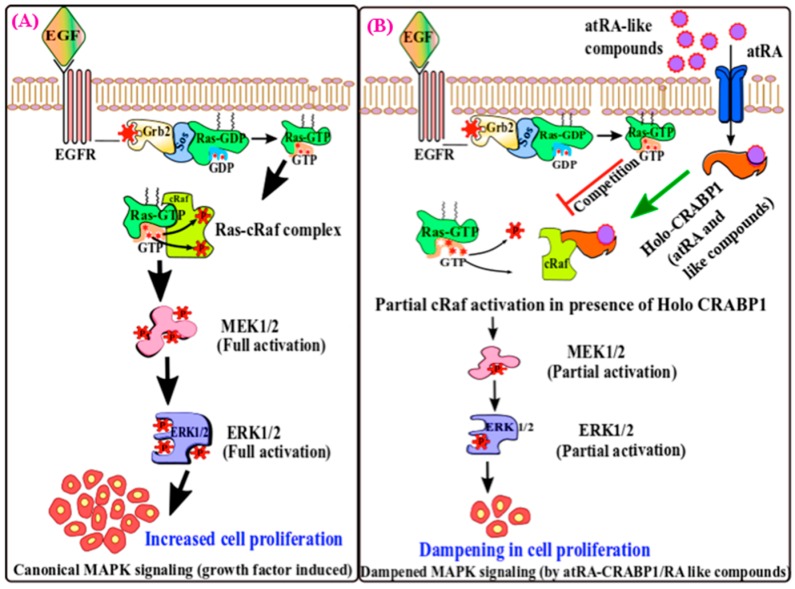
A model illustrating the effect of atRA-CRABP1 in blunting growth activation in stem cells. (**A**) In response to growth factors like EGF, robust canonical MAPK signaling is triggered involving the Ras-mediated activation of cRAF kinase by protein–protein interaction, which leads to sequential phosphorylation of MEK1/2 and ERK1/2 and enhances cell proliferation. (**B**) Cells co-exposed to EGF and atRA show reduced MAPK signaling by competitive binding of atRA-CRABP1 with cRAF, resulting in weak Ras-cRAF protein–protein interactions and downstream ERK1/2 phosphorylation, thus dampening cellular growth.

Studies using *CRABP1* knockdown ESCs have provided unambiguous evidence that phase 1 ERK1/2 activation by atRA is CRABP1-dependent [26]. This phenomenon is also observed in cancer cells using gene knockdown approaches [71]. Mechanically, atRA elicits such a non-canonical activity via CRABP1 [75], which directly interacts with rapidly accelerated fibrosarcoma (RAF), the first kinase typically activated by growth factors. This modulates the extent of the classical activation of ERK1/2 in ESCs by growth factors, such as in the presence of epidermal growth factor (EGF) that binds to EGF receptor (EGFR). Stimulated EGFR then activates Ras GTPase, which further activates RAF kinase [76,77]. Once activated, RAF phosphorylates mitogen-activated protein kinase MAPK-ERK kinase 1/2 (MEK1/2), which then phosphorylates/activates ERK1/2 [78]. In propagating this classical growth factor signaling pathway, the Ras-binding domain (RBD) of RAF plays an important role by engaging critical protein–protein interactions that regulate RAF activity [79]. In this context, CRABP1 functions by binding to the RBD of RAF, thereby modulating the activation of cRAF. NMR studies demonstrated that CRABP1 directly interacts with the RBD of cRAF and that atRA regulates this interaction, thereby negatively modulating growth-factor-stimulated RAF activation and its downstream signaling, leading to dampened ERK1/2 activation [75]. Thus, atRA, via binding to CRABP1, provides a physiological check (or dampening signal) for growth-factor-stimulated RAF activation in stem cells, thereby reducing their sensitivity to growth factor stimulation (Figure 1). This mechanism may operate in the context of the maintenance of a healthy stem cell population, which is to avoid accidental, unwanted stimulation by growth factors.

**Figure 2:**
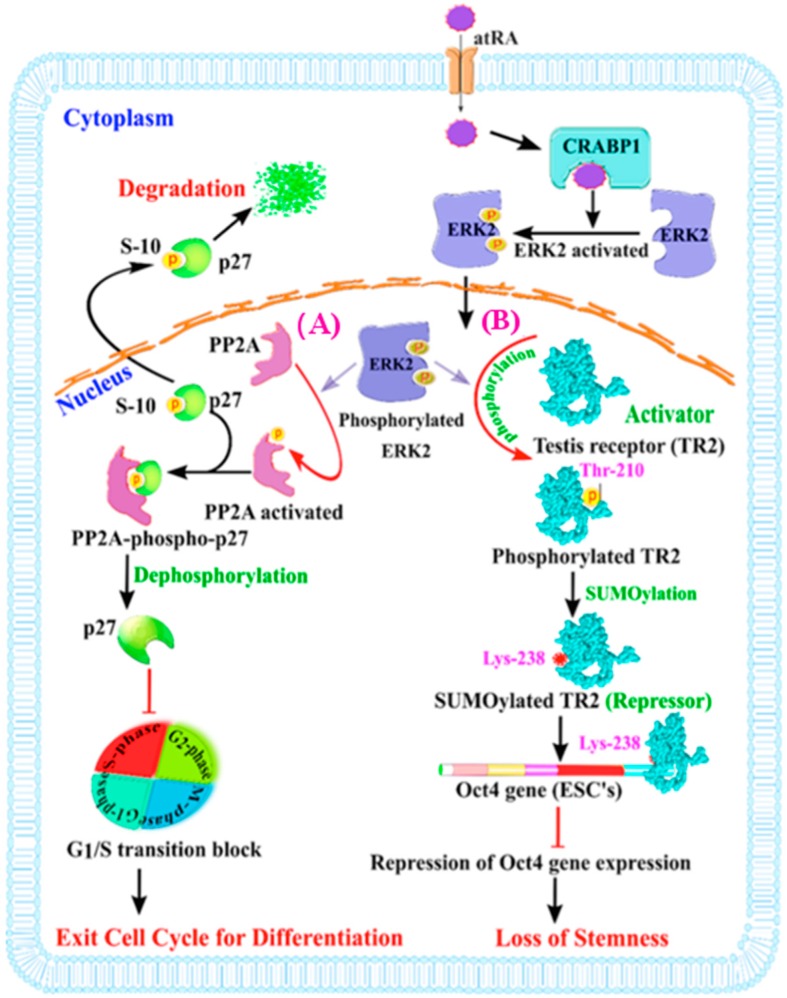
Schematic illustration of atRA-CRABP1 that activates ERK1/2 in stem cells when the level of growth factors is lowered, which dampens stem proliferation and facilitates differentiation. In the cytoplasm, atRA binds to CRABP1 and promotes MEK/ERK1/2 complex formation and activation of ERK1/2, which then translocates into the nucleus to (**A**) induce dephosphorylation of p27 (stabilized) at Ser-10 by activating PP2A and impede G1/S transition and facilitate stem cells differentiation; or (**B**) phosphorylate TR2 at Thr-210, promoting pml (promyelocytic leukemia) recruitment and SUMOylation of TR2, which represses the transcription of *Oct4* gene and induces a loss of the stemness feature of stem cells.

A physiological consequence of modulating ERK1/2 activation by atRA-CRABP1 in stem cells can also play out in at least two cellular processes when stem cells are meant to differentiate in an environment where the level of growth factor tends to subside. The first is related to cell cycle control via regulating p27 activity [26], and the second is to stimulate the phosphorylation (at Thr-210) of the testis receptor 2, TR2 (an orphan receptor), which triggers its SUMOylation/repression, thereby repressing the expression of octamer-binding transcription factor 4 (*Oct4*), the key stemness gene in maintaining a stem cell population [23,80]. ESCs have a relatively short G1 phase, which is required for their rapid entry into S phase and continuous self-renewal [81]. Molecularly, a slender G1 phase is ensured by the degradation of the p27 protein, an important regulatory protein of the cell cycle [82,83]. p27 phosphorylation/dephosphorylation status regulates its activity. The dephosphorylated p27 physically interacts with, and inhibits, G1 CDK/cyclin complexes (cyclin-dependent kinases), thus blocking the G1 to S phase transition [84]. When p27 is phosphorylated, particularly at ser-10, it is exported to cytosol for degradation [82,85]. In ESCs, when growth factors are withdrawn or reduced, atRA-CRABP1 can activate ERK1/2, which then rapidly translocates into the nucleus and phosphorylates/activates protein phosphatase 2A (PP2A) [26,71], which in turn enhances the stability of the p27 protein by dephosphorylation, thereby blocking G1 CDK/cyclin complexes and dampening cellular growth (Figure 2A, left).

The second process affected by ERK1/2 in differentiating stem cells is related to the maintenance of their stemness (Figure 2B, right). To ensure continuous stem cell proliferation, a proper amount of the stemness gene product, *Oct4*, is essential [80,86]. *Oct4* expression is regulated mainly by positive and negative transcriptional controls, where TR2 orphan nuclear receptor’s activity is important [23,80,87]. *Oct4* gene transcription is fine-tuned by the homeostasis of multiple activators and repressors. The activators include polymerase-associated factor 1 (PAF1) complex [88,89], non-POU domain-containing octamer-binding (NonO) protein [90], steroidogenic factor-1 (SF-1) [91], TR2 [80], etc. The key repressors are SUMOylated TR2 [80] and chicken ovalbumin upstream promoter transcription factors (COUP-TFs) or Pou5f1 [92]. In this context, atRA-CRABP1-stimulated ERK1/2 initiates TR2 phosphorylation at Thr-210 and its subsequent SUMOylation at Lys238, resulting in the replacement of coactivator p300/CBP associated factor (Pcaf) by corepressor RIP140, which turns TR2 from an activator to a repressor of *Oct4* gene transcription [80]. Therefore, for differentiating ESCs, atRA’s non-canonical activity rapidly activates ERK1/2, via CRABP1, and contributes to the rapid repression of the *Oct4* gene and timely suspension of their stemness feature, allowing differentiation to efficiently occur.

Together, for stem cells, the non-canonical effects of atRA’s, mediated by CRABP1, provide a timely and rapid control that not only desensitizes stimulation by growth factors (by intercepting the RAF/MEK/ERK axis) (Figure 1), but also stimulates their differentiation by augmenting cell cycle control and the dampening stemness feature (Figure 2B, right). This non-canonical, rapid activity of atRA may help to ensure that the genomic effects of atRA, that typically manifest later by RAR-mediated transcriptional events, can effectively take place in order to induce cell differentiation.

In a more physiologically relevant stem cell context, such as maintaining the NSC pool in the brain, the non-canonical activity of atRA via CRABP1 appears to be important in maintaining the NSC pool in the hippocampus [70]. In a *CRABP1* gene KO mouse brain, the NSC pool is expanded due to the removal of CRABP1 that otherwise provides a negative modulation for NSC proliferation efficiency. As a result of NSC expansion in the hippocampus, neurogenesis in the *CRABP1* gene KO mice is improved, and these *CRABP1* gene KO mice indeed exhibit behavioral changes in line with improved learning and memory [70].

### 3.2. *AtRA-CRABP1* Modulates CaMKII and Cardiomyocyte Function

AtRA-CRABP1 is also found to modulate CaMKII activation in certain differentiated cells such as cardiomyocytes [72,93]. CaMKII is critical for proper heart function, such as contraction, and also contributes to cardiomyocyte death, particularly in heart failure [94]. Targeting β-adrenergic receptors (β-AR) by agonist catecholamines, such as isoproterenol (ISO), is one of the commonly used clinical practices in treating cardiac disease [95]. However, sustained and/or excessive stimulation of CaMKII by catecholamines causes cardiac remodeling, hypertrophy, and cardiac failure [96,97]. CaMKII is a protein kinase capable of phosphorylating diverse substrates, including those leading to apoptosis and hypertrophy [94]. It appears that CRABP1 also directly interacts with CaMKIIδ (a predominant isoform of CaMKII) in the heart, thereby competing with calmodulin (CaM) to dampen Ca^2+^ CaM-induced CaMKII activation [72].

In mice, deleting CRABP1 leads to spontaneously developed, reduced cardiac function in older animals (such as a decrease in the ejection fraction of the left ventricle), corresponding to spontaneous over-activation of CaMKII in cardiomyocyte. Further, under acute ISO treatment, *CRABP1* gene KO mice showed more severe cytotoxicity as compared to wild type (WT) mice. Under a chronic ISO challenge, *CRABP1* gene KO mice exhibited a more severe cardiac phenotype, as reflected in a further enlarged heart and cardiomyocyte hypertrophy and fibrosis. Mechanically, CRABP1 can directly interact with CaMKII to inhibit its activation, and the addition of atRA further enhances this interaction. Therefore, without CRABP1, CaMKII tends to be overactivated, even under an otherwise normal stimulation such as physiological signal catecholamines. Furthermore, we have recently shown that atRA can protect ISO-induced cardiac damage in WT, but not *CRABP1* gene KO mice [72]. Thus, in the physiological context of maintaining heart health, atRA-CRABP1 may also provide a mechanism to ensure the homeostasis of CaMKII activation and to protect against accidental, unwanted overactivation of CaMKII in cardiomyocyte that may lead to cardiac damage.

### 3.3. Other Non-Canonical Activities of RA

There are a few reports also demonstrating the non-genomic action of RA. For instance, atRA directly interacts with and activates cytosolic FABP5, a fatty acid binding protein that can shuttle between the cytoplasm and the nucleus. Activated FABP5 then activates nuclear receptors PPARβ/δ and thus enhances PPARβ/δ mediated gene expression [98]. Recently, another study showed that a pool of RARα is localized in the membrane lipid rafts along with G protein alpha Q (Gαq). Intriguingly, the efficiency of the RARα-Gαq complex prominently increased upon treatment with RA. This finding indicates that the RARα-Gαq complex can exist in the cytoplasm, which can be enhanced by cytoplasmic RA [99]. Gαq is an activator of p38 MAPK; thus, by increasing the pool of the RARα-Gαq complex, RA can enhance the activation of the p38 MAPK pathway without triggering a genomic effect [100]. However, the study did not provide compelling genetic evidence, casting doubt over the physiological relevance of this intriguing signaling pathway.

The emerging “non-canonical” signaling is also supported by additional findings about the potential signaling cascade of retinoid, the alcohol form of retinoid. It appears that the retinol-retinol binding protein (RBP) complex can bind and activate cell surface receptor signaling cascades by forming a STRA6-RBP complex to regulate the JAK-STAT signaling pathways [51,101].

## 4. Conclusions and Future Directions

AtRA is the principal active metabolite of vitamin A and represents one classical endocrine hormone, which mainly elicits genomic effects. This type of canonical signaling typically spans a more extended period of time and causes more permanent changes in cellular processes often associated with an alteration in gene expression. With this principal action, atRA is known primarily as a differentiation agent. Recent studies present evidence for atRA’s new activities, mediated by its cytoplasmic binding protein CRABP1, but not by its nuclear receptors RARs/RXRs, to elicit very rapid (within minutes) activities intercepting several specific cytosolic signaling pathways such as growth factor-stimulated ERK1/2 (for stem cells) and catecholamine-triggered CaMKII activation (for cardiomyocytes). While results of gene KO studies unambiguously demonstrate the consequences of deleting these specific pathways, both in animals and in primary cells, the physiological context of this non-canonical signaling remains to be further elucidated. For instance, questions remain to be answered when it comes to the integration of atRA (or vitamin A status) with other growth factors or neuropeptides in the context of whole animals. Will hypovitaminosis A also cause an abnormal NSC pool in the brain or more severe cardiac outcome? Given that CRABP1 can intercept growth factor signaling, can CRABP1 also serve as a target in cancer therapy? Classical transgenic studies of over-expressing *CRABP1* demonstrate that abnormally high levels of *CRABP1* can lead to abnormalities in the lung and the liver in mice [68]. Does this contribute to the pathology of hypervitaminosis A? Among all receptors and binding proteins for RA, CRABP1 is the most conserved member. This also suggests a versatile physiological role for CRABP1, which constrains the progression in its molecular divergence during evolution. Another important question is, can other forms of RA also elicit non-canonical activity via binding CRABP1? Finally, it is tempting to speculate that there remain more functions of RA-CRABP1 to be identified.

To further dissect these functions, there is an urgent need to determine other CRABP1- interacting partners/signaling networks. One such approach can be through utilizing molecular docking to identify certain atRA-like compounds that can mimic atRA’s action by binding to CRABP1. Because such compounds are less likely to produce the canonical effects of atRA (which requires binding to RARs), studies using such compounds can provide a better insight into CRABP1′s crosstalk with other signaling pathways without interference from RAR/RXR-mediated nuclear activities of RA. Further, protein-protein docking of CRABP1, followed by experimental evaluation, can reveal additional potential partner proteins of CRABP1 and their functional roles in other signaling pathways. Finally, from a therapeutic point of view, compounds specific to CRABP1 that do not bind to RARs/RXRs would be better candidates as therapeutics for targeting CRABP1 to selectively intercept certain biological processes, such as inhibiting cancer cell growth and improving survival/function.
